# Prognostic and Genomic Analysis of Proteasome 20S Subunit Alpha (PSMA) Family Members in Breast Cancer

**DOI:** 10.3390/diagnostics11122220

**Published:** 2021-11-27

**Authors:** Chung-Chieh Chiao, Yen-Hsi Liu, Nam Nhut Phan, Nu Thuy An Ton, Hoang Dang Khoa Ta, Gangga Anuraga, Do Thi Minh Xuan, Fenny Fitriani, Elvira Mustikawati Putri Hermanto, Muhammad Athoillah, Vivin Andriani, Purity Sabila Ajiningrum, Yung-Fu Wu, Kuen-Haur Lee, Jian-Ying Chuang, Chih-Yang Wang, Tzu-Jen Kao

**Affiliations:** 1 Ph.D. Program for Cancer Molecular Biology and Drug Discovery, College of Medical Science, Taipei Medical University, Taipei 11031, Taiwan; D621110004@tmu.edu.tw (C.-C.C.); d621109004@tmu.edu.tw (H.D.K.T.); g.anuraga@unipasby.ac.id (G.A.); khlee@tmu.edu.tw (K.-H.L.); 2 Graduate Institute of Cancer Biology and Drug Discovery, College of Medical Science and Technology, Taipei Medical University, Taipei 11031, Taiwan; M120110028@tmu.edu.tw (Y.-H.L.); m654110001@tmu.edu.tw (D.T.M.X.); 3 NTT Institute of Hi-Technology, Nguyen Tat Thanh University, Ho Chi Minh City 700000, Vietnam; pnnam@ntt.edu.vn (N.N.P.); tntan@ntt.edu.vn (N.T.A.T.); 4 Department of Statistics, Faculty of Science and Technology, Universitas PGRI Adi Buana, Surabaya 60234, Indonesia; fenny_f@unipasby.ac.id (F.F.); elvira@unipasby.ac.id (E.M.P.H.); athoillah@unipasby.ac.id (M.A.); 5 Department of Biological Science, Faculty of Science and Technology, Universitas PGRI Adi Buana, Surabaya 60234, Indonesia; v.andriani@unipasby.ac.id (V.A.); puritysabila@unipasby.ac.id (P.S.A.); 6 Department of Medical Research, Tri-Service General Hospital, School of Medicine, National Defense Medical Center, Taipei 11490, Taiwan; qrince@yahoo.com.tw; 7 Cancer Center, Wan Fang Hospital, Taipei Medical University, Taipei 11031, Taiwan; 8 TMU Research Center of Cancer Translational Medicine, Taipei Medical University, Taipei 11031, Taiwan; chuangcy@tmu.edu.tw; 9 Ph.D. Program for Neural Regenerative Medicine, Taipei Medical University, Taipei 11031, Taiwan; 10 Research Center of Neuroscience, Taipei Medical University, Taipei 11031, Taiwan

**Keywords:** *PSMA* family genes, bioinformatics, breast cancer

## Abstract

The complexity of breast cancer includes many interacting biological processes, and proteasome alpha (PSMA) subunits are reported to be involved in many cancerous diseases, although the transcriptomic expression of this gene family in breast cancer still needs to be more thoroughly investigated. Consequently, we used a holistic bioinformatics approach to study the *PSMA* genes involved in breast cancer by integrating several well-established high-throughput databases and tools, such as cBioPortal, Oncomine, and the Kaplan–Meier plotter. Additionally, correlations of breast cancer patient survival and PSMA messenger RNA expressions were also studied. The results demonstrated that breast cancer tissues had higher expression levels of *PSMA* genes compared to normal breast tissues. Furthermore, *PSMA2*, *PSMA3*, *PSMA4*, *PSMA6*, and *PSMA7* showed high expression levels, which were correlated with poor survival of breast cancer patients. In contrast, *PSMA5* and *PSMA8* had high expression levels, which were associated with good prognoses. We also found that *PSMA* family genes were positively correlated with the cell cycle, ubiquinone metabolism, oxidative stress, and immune response signaling, including antigen presentation by major histocompatibility class, interferon-gamma, and the cluster of differentiation signaling. Collectively, these findings suggest that *PSMA* genes have the potential to serve as novel biomarkers and therapeutic targets for breast cancer. Nevertheless, the bioinformatic results from the present study would be strengthened with experimental validation in the future by prospective studies on the underlying biological mechanisms of *PSMA* genes and breast cancer.

## 1. Introduction

According to the most up-to-date statistics provided by the World Health Organization (WHO), the progressively increasing estimates of cancer cases and deaths in recent years make it one of the leading causes of premature deaths worldwide [1]. Although the cancer incidences and mortality rates vary significantly among ages, genders, and regions, it is indisputable that the cancer burden rises with age and population increment, particularly in China, North America, and Europe. Although lung cancer by far remains the leading killer of both sexes, it was recently displaced by breast cancer (BRCA) in terms of the most frequently diagnosed cases among women. Early detection and appropriate treatment are believed to play crucial roles in reducing the immense health and economic burdens imposed and extending the life expectancy for cancer survivors in general and particularly in terms of BRCA due to its invasiveness and aggressiveness. Compared to early and locally advanced BRCA, which is considered curable, advanced stages and metastatic BRCA, along with inflammatory BRCA (IBC) and triple-negative BRCA (TNBC), lack druggable molecular targets, thus resulting in limited treatment regimens [2,3,4]. Therefore, unremitting efforts have been made to enhance the precision of the prognostic predictions of female BRCA; among these, molecular signatures and targeted therapies have emerged as promising treatment regimens of distinct subtypes [5].

While existing evidence-based treatment strategies use the classical hormonal factors, including the progesterone receptor (PR), estrogen receptor (ER), and human epidermal growth factor receptor (HER)-2, to stratify BRCA prior to determining the most suitable treatment for patients, plenty of immunohistochemical markers, such as Ki-67, p53, and E-cadherin, are simultaneously employed as predictive tools for those subtypes that still lack druggable molecular targets [6,7,8,9,10]. Over the past few years, studies of the genetic alterations and dysfunction of signal transduction pathways that highly contribute to the advent of numerous predictive biomarkers, including transcriptomic data and messenger (m)RNA levels, have opened up the possibility of having effective therapeutics and have been useful in predicting tumor grades, drug responsiveness, and risks of recurrence of intrinsic subtypes [11,12,13].

The degradation of obsolete or damaged endogenous proteins is catalyzed by the 26S proteasome, which is comprised of two complexes, namely 19S (the regulatory complex) and 20S (the core complex) [14,15]. There is a growing body of studies regarding the involvement of proteases in various critical cellular processes of cancer cells, such as proliferation, apoptosis, the cell cycle, DNA repair, invasion, and metastasis [16,17,18]. Within the 20S core complex, proteasome alpha (PSMA) subunits are the main subunits, which are constituted of eight unique alpha subunits (PSMA1~8). Associations of these *PSMA* genes with cancers have been documented [19]. For instance, significant increases in *PSMA1* and *PSMA5* were found in pulmonary neuroendocrine tumors relative to normal tissues [20]. The previous literature also demonstrated implications of proteasome subunit genes in several cancerous diseases, namely BRCA, lung cancer, hepatocellular carcinoma (HCC), and colorectal cancer (CRC). Of the proteasome genes, *PSMB5* and *PSMD10* were reported to be associated with the proliferation of TNBC and to promote the invasiveness and metastasis of HCC [21,22,23].

To study the roles of *PSMA* family genes in BRCA, it is worth comprehensively screening their interactions in an integrated model using a holistic approach. To implement this high-throughput analysis, we combined multiple tools and databases containing transcriptomic expressions of *PSMA* genes. Integrating multiple datasets from these databases can provide a high degree of evidence from various studies in different populations using either raw or processed data, such as the Gene Expression Omnibus (GEO) (https://www.ncbi.nlm.nih.gov/geo/ accessed on 1 February 2021), a biomedical repository providing transcriptomic datasets for both microarray and next-generation sequencing platforms [24,25,26,27,28,29,30,31,32]. Changes in the mRNA expression levels of genes, sometimes an order of magnitude higher or lower in tumors than in normal matched tissues, illustrate, respectively, their roles as either oncogenes or tumor suppressors in cancer [33,34,35]. Applying this concept to BRCA and *PSMA* family genes, we used multiple datasets from public databases to study the expressions of *PSMA* genes in multiple subtypes of BRCA together with interaction networks to trace potential coexpressed targets with *PSMA* genes.

## 2. Materials and Methods

### 2.1. UALCAN Analyses

Transcriptomic expressions of *PSMA* members were analyzed in BRCA samples using the UALCAN (http://ualcan.path.uab.edu/ accessed on 1 February 2021) platform. UALCAN collected the Cancer Genome Atlas (TCGA) level 3 RNA-Seq as well as the patients data from different cancer types. With genes of interest, UALCAN allows users to identify biomarkers to verify gene expressions with multiple clinical factors. We created a boxplot of *PSMA* mRNA expression levels measured in BRCA specimens (red) compared to their normal counterparts (blue) obtained from the UALCAN database, and used Student’s *t*-test to calculate the significant differences between groups with a *p*-value threshold of 0.01 [36].

### 2.2. Kaplan–Meier (KM) Plot of Survival Analysis

To explore transcriptomic effects of *PSMA* gene family members to relapse-free survival (RFS) of BRCA patients, a survival analysis using the KM plotter database (https://kmplot.com/ accessed on 1 February 2021) was conducted. The breast cancer patients (n = 4929) were collected from the Gene Expression Omnibus (GEO) and TCGA (HG-U133A 2.0, Affymetrix HG-U133A, and HG-U133 Plus 2.0 microarrays) as previously described [37,38]. The KM plotter was applied with default settings for the survival analysis, including log-rank *p* values, with the Jet set as the best probe set, and hazard ratios (HRs) with 95% confidence intervals (CIs). Log-rank *p* < 0.05 was considered statistically significant.

### 2.3. Analysis of Protein Expressions in Human Clinical Specimens

The Human Protein Atlas (HPA, www.proteinatlas.org accessed on 1 February 2021) provides a wealth of information on sequences, pathologies, expressions, and distributions in various cancer tissues. The first version of this database contained more than 400,000 high-resolution photo corresponding to more than 700 antibodies to human proteins [39,40,41]. This study analyzed the differential statuses of protein expressions and localization of select members of the PSMA family protein expression in breast tissues [42,43].

### 2.4. Functional Enrichment Analysis of PSMA Family Members

We extracted data from the METABRIC and TCGA datasets in the cBioPortal (https://www.cbioportal.org accessed on 1 February 2021) database to analyze functional enrichment, and further used the MetaCore Analysis (https://portal.genego.com accessed on 1 February 2021) to explore the downstream network, and *p* < 0.05 was set as the boundary criterion as we previously described [44,45,46,47,48].

### 2.5. DNA Methylation

To assess the methylation status of a target gene, we utilized Methsurv (https://biit.cs.ut.ee/methsurv/ accessed on 1 February 2021) to produce a heatmap of the various DNA methylated areas [49]. To illustrate DNA methylation levels, beta values were employed (ranging from 0 to 1). The beta value for each CpG site is calculated using M/ (M + U + 100). The methylated and unmethylated intensities are represented by M and U, respectively.

## 3. Results

### 3.1. PSMA Family Members Play Crucial Roles in BRCA Development

**Figure 1:**
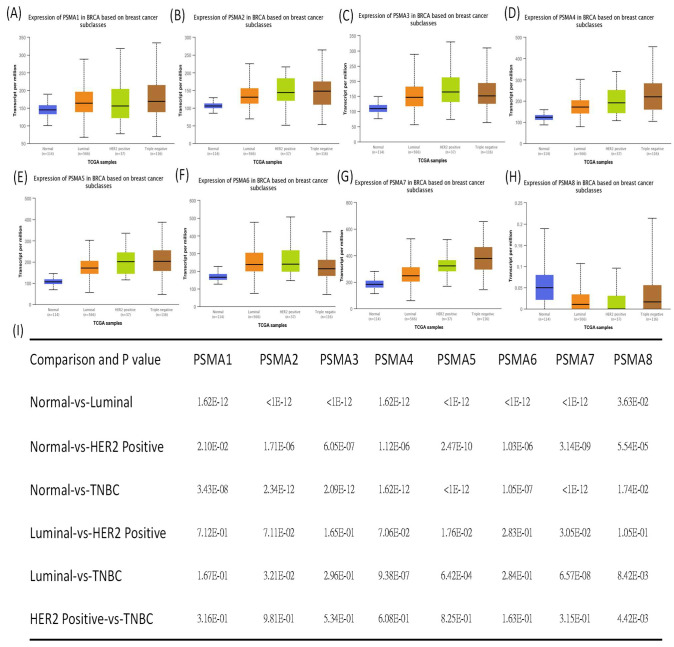
Proteasome 20S subunit alpha (*PSMA*) gene family transcription levels in different stages of breast cancer (BRCA) patients (UALCAN analysis). (**A**–**H**) Box plot of *PSMA* gene family and primary tumor (BRCA) tissues. The box plot shows comparisons of the expressions of TCGA data from the *PSMA* gene family in different stages of breast cancer, including normal samples (*n* = 114), luminal (*n* = 566), HER2-positive (*n* = 37), and triple-negative tumors (*n* = 116). Statistical significance is represented by *p* < 0.05. (**I**) The table shows relative expression levels of *PSMA* family genes in normal samples and different BRCA subtypes; *p* < 0.05 was considered statistically significant.

**Figure 2:**
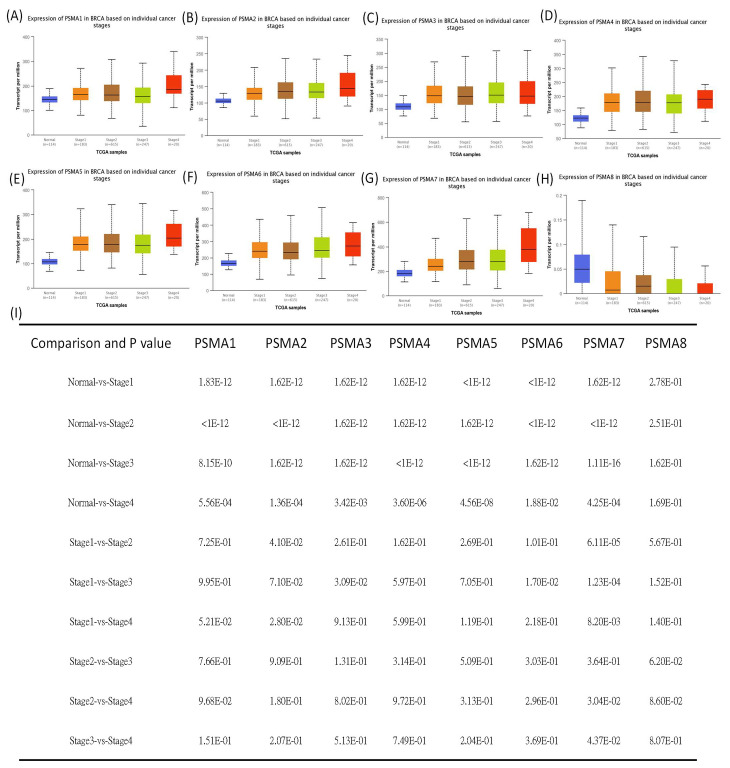
Proteasome 20S subunit alpha (*PSMA*) family gene transcription levels in different stages of breast cancer patients. (**A**–**H**) Box plot shows relative expression levels of *PSMA* family genes in normal samples (*n* = 114), and stage 1 (*n* = 183), stage 2 (*n* = 615), stage 3 (*n* = 247), and stage 4 breast cancer (*n* = 20). (**I**) The table shows relative expression levels of *PSMA* family genes in different BRCA stages; *p* < 0.05 was considered statistically significant.

The previous literature reported that several members of the *PSMA* gene family were involved in cancer onset and development. Therefore, studying all of these PSMA members in BRCA is an essential approach that would provide detailed evidence suggesting their potential roles as biomarkers for BRCA subtypes. The UALCAN analysis found that the *PSMA1~7* genes had significantly higher transcriptomic expression levels in BRCA tissues than normal tissues, which was contradictory to the results for the *PSMA8* gene (Figure 1). It was noted that, in further analyses of the expressions of *PSMA* family genes in BRCA patients, their expression levels were highly correlated with tumor grades, tumor stages, and metastatic events (Figure 2).

### 3.2. Protein Expression Levels and Prognostic Values of PSMA Family Members in BRCA Specimens

**Figure 3:**
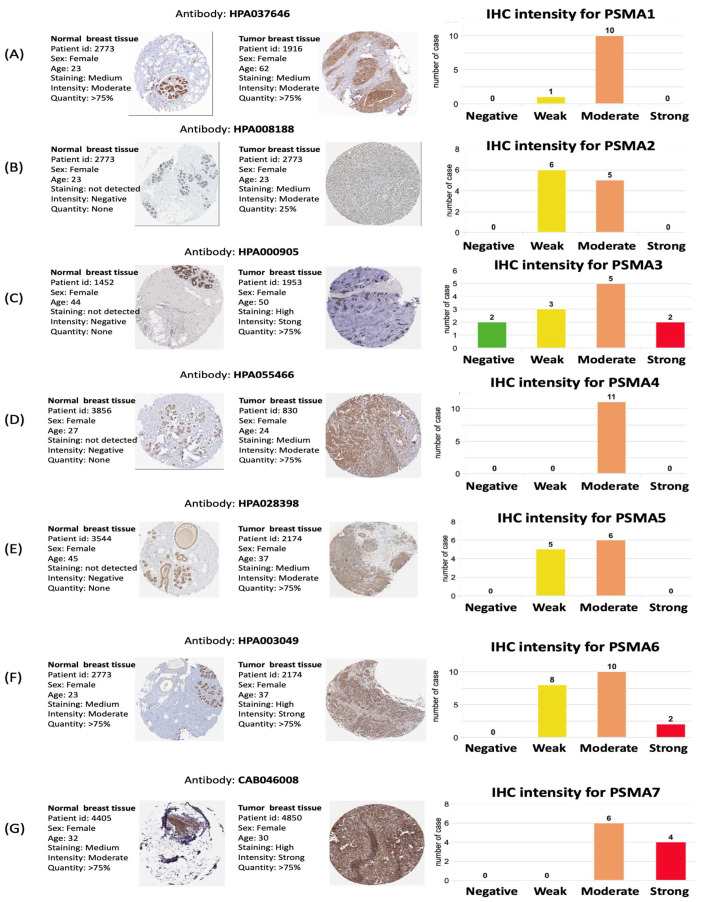
Protein expression levels of proteasome 20S subunit alpha (PSMA) family members across clinical breast cancer specimens from the Human Protein Atlas. (**A**–**G**) PSMA3, PSMA6, and PSMA7 showed high protein expression levels, and most PSMA family members showed medium protein expression levels in breast cancer specimens. All IHC images and patient information were acquired from the Human Protein Atlas.

**Figure 4:**
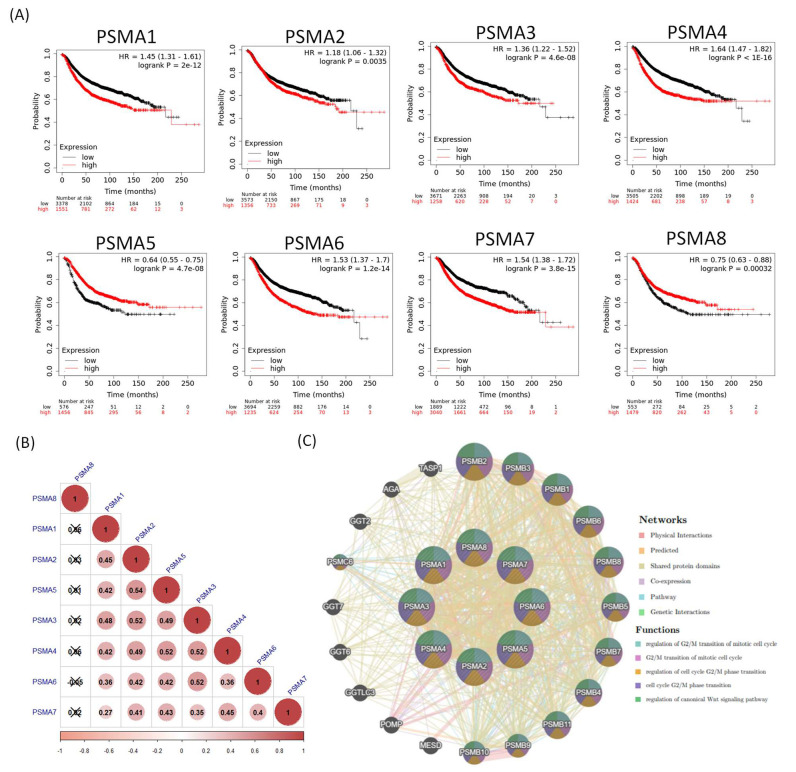
Prognosis and correlation analysis of proteasome 20S subunit alpha (*PSMA*) family genes in breast cancer patients. (**A**) A recurrence-free survival (RFS) dataset was used for the analysis. An auto-cutoff was applied in this analysis to differentiate patients into two groups based on the best cutoff value of PSMA mRNA. Higher and lower expression levels of PSMA mRNA than the cutoff value are, respectively, indicated in red and black. Significant correlations were shown between high expression levels of PSMA1, PSMA2, PSMA3, PSMA4, PSMA6, and PSMA7 with poor survival outcomes in breast cancer patients (n = 4929). (**B**) Correlations between *PSMA* family genes in breast cancer patients, and insignificant correlations are marked by crosses. (**C**) Co-regulated molecules for *PSMA* family genes were analyzed with the GeneMania platform.

Together with a transcriptomic expression analysis of *PSMA* genes, we further carried out an analysis at the proteomic level in clinical human BRCA specimens. This could potentially provide interesting correlations between *PSMA* genes and BRCA subtypes through antibody intensity levels. The protein expression levels of PSMA family members and their clinical relevance were determined by analyzing the Human Protein Atlas (HPA) database. PSMA3, PSMA6, and PSMA7 were found to have high protein expression levels, and most PSMA family members showed medium protein expression levels in BRCA specimens (Figure 3). The KM plot of this database also showed that PSMA1, PSMA2, PSMA3, PSMA4, PSMA6, and PSMA7 had high expression levels in BRCA tissues relative to normal breast cells and predicted poor survival, whereas PSMA5 and PSMA8 did not. These data implied that most PSMA family members might have oncogenic roles in BRCA progression (Figure 4A). We also investigated mRNA expression correlations among *PSMA* gene family members (Figure 4B) and co-regulated molecules (Figure 4C). Meanwhile, DNA methylation is an epigenetic modification that has been involved in the formation of several malignancies. We present a heatmap of DNA methylation grouping the expression levels of the *PSMA* gene family in breast cancer, as well as its predictive relevance. Among the *PSMA* family genes, we observed that cg07435350, cg26165081, cg26868250 of *PSMA1*; cg10778455, cg106226670, cg15202134 of *PSMA2*; cg08095532, cg14211735 of *PSMA4*; cg08250978, cg13170147 of *PSMA5*; cg01757308 of *PSMA6*; cg17665883 of *PSMA7*; cg11858305, cg15865827, cg00262344, cg06377543, cg03162994, cg22027766, cg259833544, cg01070760, cg21248196 of *PSMA8* indicated the highest level of DNA methylation in breast cancer (Figure S1).

### 3.3. Pathway and Network Analysis of PSMA Family Genes

**Figure 5:**
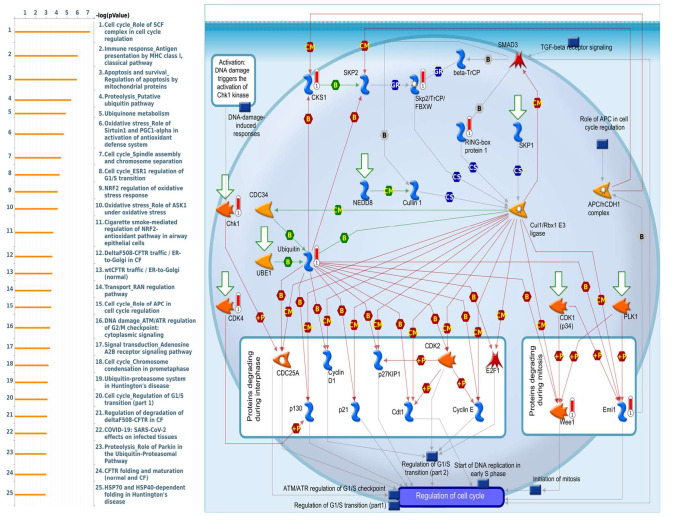
MetaCore pathway analysis of the coexpression gene network of proteasome 20S subunit alpha 1 (PSMA1) in breast cancer patients. We used the MetaCore platform to analyze genes coexpressed with PSMA1 from the associated METABRIC and TCGA datasets, and downstream pathway analyses revealed that “Cell cycle_Role of SCF complex in cell cycle regulation” participates in breast cancer development.

**Figure 6:**
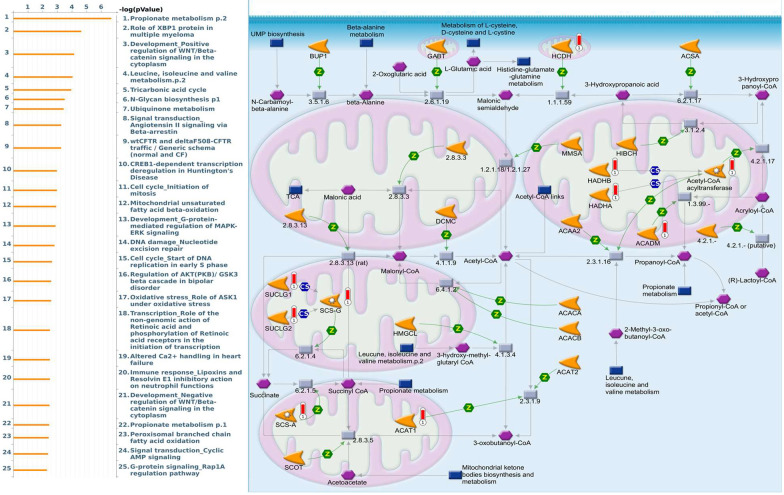
MetaCore pathway analysis of the coexpression gene network of proteasome 20S subunit alpha 2 (PSMA2) in breast cancer patients. We used the MetaCore platform to analyze genes coexpressed with PSMA2 from the associated METABRIC and TCGA datasets, and downstream pathway analyses revealed that “Propionate metabolism p.2” participates in breast cancer development.

**Figure 7:**
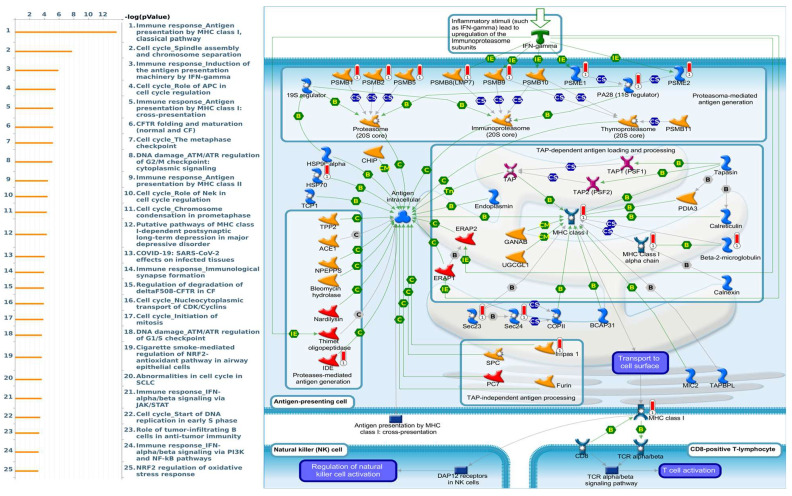
MetaCore pathway analysis of the coexpression gene network of proteasome 20S subunit alpha 3 (PSMA3) in breast cancer patients. We used the MetaCore platform to analyze genes coexpressed with PSMA3 from the associated METABRIC and TCGA datasets, and downstream pathway analyses revealed that “Immune response_Antigen presentation by MHC class I, classical pathway” participates in breast cancer development.

**Figure 8:**
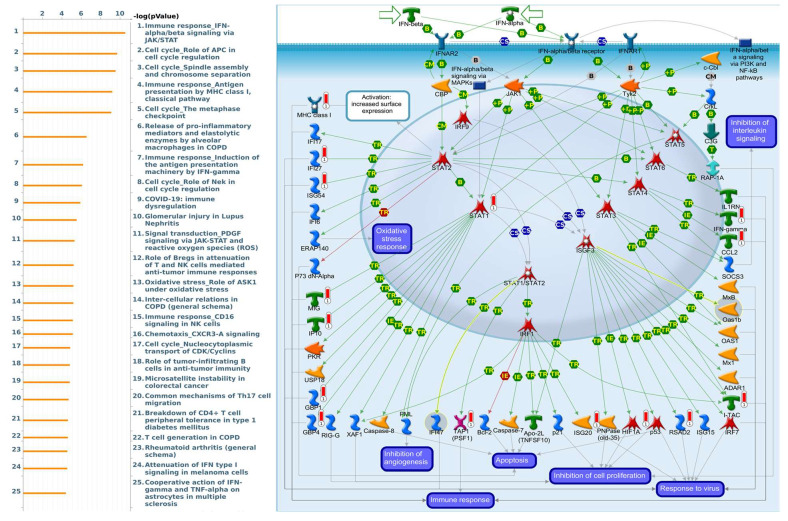
MetaCore pathway analysis of the coexpression gene network of proteasome 20S subunit alpha 4 (PSMA4) in breast cancer patients. We used the MetaCore platform to analyze genes coexpressed with PSMA4 from the associated METABRIC and TCGA datasets, and downstream pathway analyses revealed that “Immune response_IFN-alpha and beta signaling via JAK/STAT” participates in breast cancer development.

**Figure 9:**
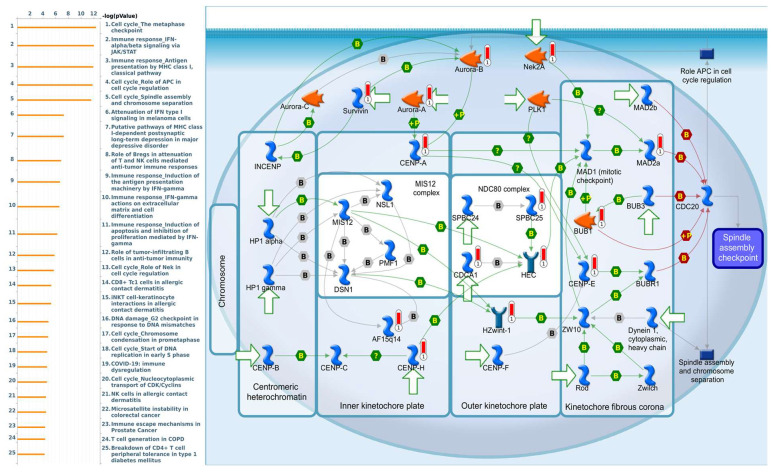
MetaCore pathway analysis of the coexpression gene network of proteasome 20S subunit alpha 5 (PSMA5) in breast cancer patients. We used the MetaCore platform to analyze genes coexpressed with PSMA5 from the associated METABRIC and TCGA datasets, and downstream pathway analyses revealed that “Cell cycle_The metaphase checkpoint” participates in breast cancer development.

**Figure 10:**
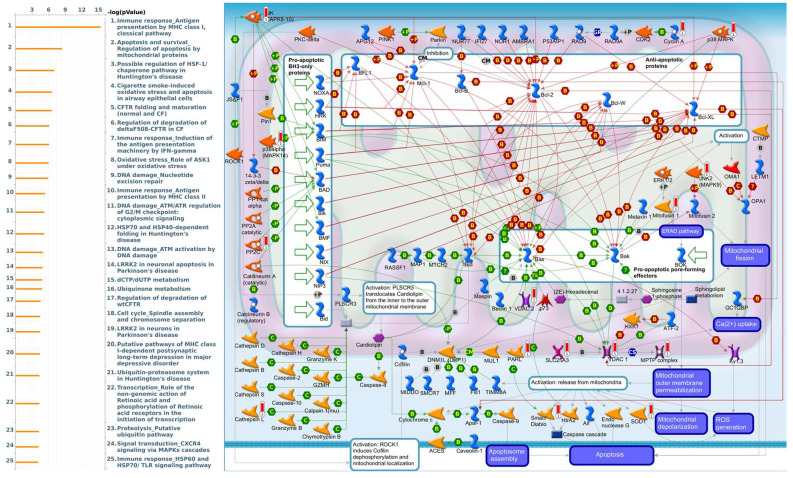
MetaCore pathway analysis of the coexpression gene network of proteasome 20S subunit alpha 6 (PSMA6) in breast cancer patients. We used the MetaCore platform to analyze genes coexpressed with PSMA6 from the associated METABRIC and TCGA datasets, and downstream pathway analyses revealed that “Apoptosis and survival_Regulation of apoptosis by mitochondrial proteins” participates in breast cancer development.

Enriched biological processes shown by GeneGo MetaCore version 21.1 (Cortellis, Philadelphia, PA, USA) software revealed that genes coexpressed with *PSMA* family genes had high correlations with cancer developmental processes. In addition, MetaCore can be used to construct biological networks specific for each tissue from gene lists. Lists of genes coexpressed with *PSMA* genes obtained from TCGA and METABRIC were input to the MetaCore platform. The results showed that these genes were associated with various signal pathways in cancer progression. The top 10% of coexpressed genes for each PSMA member were also used for enrichment analysis. We found that genes coexpressed with *PSMA1* were involved in cell cycle-related pathways and networks, such as “Cell cycle_Role of SCF complex in cell cycle regulation”, “Immune response_Antigen presentation by MHC class I, classical pathway”, “Apoptosis and survival_Regulation of apoptosis by mitochondrial proteins”, “Proteolysis_Putative ubiquitin pathway”, and “Ubiquinone metabolism” (Figure 5, Supplementary Table S1). Genes coexpressed with *PSMA2* were involved in metabolism-related pathways and networks, such as “Propionate metabolism p.2”, “Leucine, isoleucine, and valine metabolism p.2”, “Development_Positive regulation of WNT/Beta-catenin signaling in the cytoplasm”, “Tricarbonic acid cycle”, and “N-Glycan biosynthesis p.1” (Figure 6, Supplementary Table S2). Genes coexpressed with *PSMA3* were involved in immune-related pathways and networks, such as “Immune response_Antigen presentation by MHC class I, classical pathway”, “Cell cycle_Spindle assembly and chromosome separation ”, “Immune response_Induction of the antigen presentation machinery by IFN-gamma ”, “CFTR folding and maturation (normal and CF)”, and “Immune response_Antigen presentation by MHC class I: cross-presentation” (Figure 7, Supplementary Table S3). Genes coexpressed with *PSMA4* were involved in inflammation-related pathways and networks, such as “Immune response_IFN-alpha/beta signaling via JAK/STAT”, “Immune response_Antigen presentation by MHC class I, classical pathway”, “Release of proinflammatory mediators and elastolytic enzymes by alveolar macrophages in COPD”, “Immune response_Induction of the antigen presentation machinery by IFN-gamma”, and “COVID-19: immune dysregulation” (Figure 8, Supplementary Table S4) [50,51,52,53]. Genes coexpressed with *PSMA5* were involved in cell cycle-related pathways and networks, such as “Cell cycle_The metaphase checkpoint”, “Immune response_IFN-alpha/beta signaling via JAK/STAT”, “Immune response_Antigen presentation by MHC class I, classical pathway”, “Cell cycle_Role of APC in cell cycle regulation”, and “Cell cycle_Spindle assembly and chromosome separation” (Figure 9, Supplementary Table S5). Genes coexpressed with *PSMA6* were involved in cell cycle-related pathways and networks, such as “Immune response_Antigen presentation by MHC class I, classical pathway”, “Apoptosis and survival_Regulation of apoptosis by mitochondrial proteins”, “Immune response_Induction of the antigen presentation machinery by IFN-gamma”, “Oxidative stress_Role of ASK1 under oxidative stress”, and “Immune response_Antigen presentation by MHC class II” (Figure 10, Supplementary Table S6).

**Figure 11:**
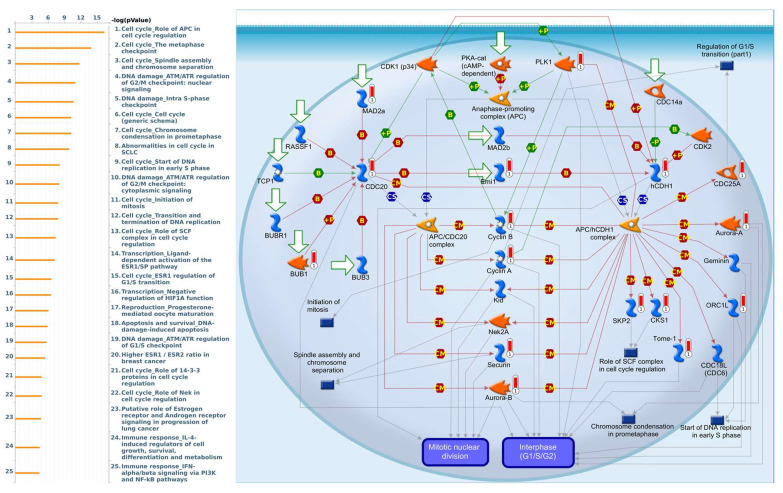
MetaCore pathway analysis of the coexpression gene network of proteasome 20S subunit alpha 7 (PSMA7) in breast cancer patients. We used the MetaCore platform to analyze genes coexpressed with PSMA7 from the associated METABRIC and TCGA datasets, and downstream pathway analyses revealed that “Cell cycle_Role of APC in cell cycle regulation” participates in breast cancer development.

**Figure 12:**
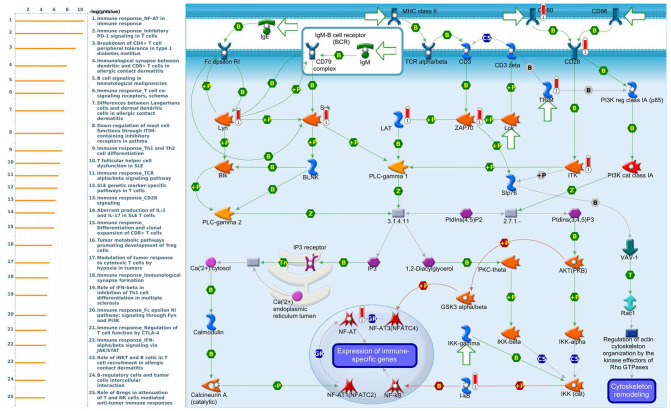
MetaCore pathway analysis of the coexpression gene network of proteasome 20S subunit alpha 8 (PSMA8) in breast cancer patients. We used the MetaCore platform to analyze genes coexpressed with PSMA8 from the associated METABRIC and TCGA datasets, and downstream pathway analyses revealed that “mmune response_NF-AT in immune response” participates in breast cancer development.

Genes coexpressed with *PSMA7* were involved in cell cycle-related pathways and networks, such as “Cell cycle_Role of APC in cell cycle regulation”, “Cell cycle_The metaphase checkpoint”, “Cell cycle_Spindle assembly and chromosome separation”, “DNA damage_ATM/ATR regulation of G_2_/M checkpoint: nuclear signaling”, and “DNA damage_Intra S-phase checkpoint” (Figure 11, Supplementary Table S7). Genes coexpressed with *PSMA8* were involved in cell immune-related pathways and networks, such as “Immune response_NF-AT in immune response”, “Immune response_Inhibitory PD-1 signaling in T cells”, “Breakdown of CD4+ T cell peripheral tolerance in type 1 diabetes mellitus”, “Immunological synapse between dendritic and CD8^+^ T cells in allergic contact dermatitis”, and “B cell signaling in hematological malignancies” (Figure 12, Supplementary Table S8).

## 4. Discussion

Recently, BRCA overtook lung cancer to rank first among the commonly occurring malignancies in women worldwide [54]. Delays in diagnosis and treatment that worsen the patient’s outcomes, along with the enormous burdens resulting from long-term and high-cost therapies, have once again undisputedly driven BRCA to become a major public health concern for scientists [55,56,57,58,59]. Clinical practice reveals that the heterogeneity of this disease, observed in both staging systems and histopathologic classification based on molecular standpoints, may complicate the accurate stratification and challenge the selection of respective therapeutic strategies [60,61,62,63]. Despite years of extraordinary efforts to enhance our knowledge of its biology and improve surgical treatments and chemotherapies, patient prognoses with advanced BRCA have not substantially improved. Due to deeper insights gained by studies on molecular alterations and advances in molecular characterization and the recognition of novel biomarkers’ roles in creating more homogenous subgroups that may guide clinical decisions, determining new biomarkers to improve patient prognoses and develop effective interventions is pivotal [64,65,66,67].

We recently reported that the high levels of PSMC family members, including PSMC1, PSMC3, PSMC4, PSMC5, and PSMC6, were positively correlated with the poor survival rates of BRCA patients [45]. Peroxisome proliferator-activated receptor γ (PPARγ) is also associated with ubiquitin–proteasome-dependent degradation and response to the angiotensin II (Ang II) system [68,69,70,71,72,73,74,75,76]. However, whether *PSMA* family genes are also involved in BRCA development still needs to be more thoroughly investigated. To the extent of our knowledge, this is the first-ever report on *PSMA* family genes that provides a comprehensive overview of the genes expressed in relation to patient survival predictions in BRCA. Although the literature has separately confirmed the dysregulation of each gene of the *PSMA* family that was individually observed in various types of cancer, along with their involvement in other tumor-related issues, only *PSMA1* and *PSMA2* have so far appeared as potential candidates. *PSMA1*, alternatively known as proteasome subunit alpha type 1, encodes the α6 subunit, which makes up the outer ring of the 20S core particle. *PSMA1* was first reported to be highly overexpressed in BRCA by Deng et al., who, while profiling antibody-inducing immunogens in tumor tissues, identified *PSMA1* as a colon cancer marker [77,78]. Consistent with previous research, *PSMA2*, alternatively known as proteasome subunit alpha type 2, encodes the α6 subunit, which makes up the outer ring of the 20S core particle. Cancer-related research revealed that, besides the overexpression of *PSMA2* mRNA recorded in colorectal cancer stages one to four, as well as in ovarian malignant tumor tissues, targeting *PSMA2* by adeno-associated viral vectors was also associated with significant decreases in cell viability and apoptosis induction in basal-like BRCA [79,80,81]. *PSMA3*, alternatively known as proteasome subunit alpha type 3, encodes the α7 subunit, which makes up the outer ring of the 20S core particle. Cancer-related reports on the relation of *PSMA3* expression and malignancy are limited, including metastatic gastric cancer and cholangiocarcinoma [82,83]. *PSMA4*, alternatively known as proteasome subunit alpha type 4, encodes the α3 subunit, which makes up the outer ring of the 20S core particle. Although little is known about their roles in cancer, prominent among those were polymorphisms of *PSMA4* that play crucial roles in the responsiveness of lung cancer patients to cisplatin-based chemotherapy [84]. *PSMA5*, alternatively known as proteasome subunit alpha type 5, encodes the outer α-rings of the 20S core particle. *PSMA5* was significantly upregulated in endometrial cancer, while *PSMA6* was significantly upregulated in multiple myeloma patients and pancreatic ductal carcinoma cell models [85,86]. *PSMA6*, alternatively known as proteasome subunit alpha type 6, encodes the α1 subunit, which makes up the outer ring of the 20S core particle. *PSMA7*, alternatively known as proteasome subunit alpha type 7, encodes the α4 subunit, which makes up outer ring of the 20S core particle. *PSMA7*, through the mitogen-activated protein kinase (MAPK) pathway, promotes the proliferation and metastasis of gastric cancer [87]. *PSMA8*, alternatively known as proteasome subunit alpha type 8, encodes the α8 subunit, which makes up the outer ring of the 20S core particle. *PSMA8* is involved in many critical processes, such as histone acetylation, DNA repair, and epigenetic regulation. These cellular processes are known for their importance in keeping cells healthy and working properly [88]. This information from the previous literature on the roles of the *PSMA* gene family is consistent with the present bioinformatic analytical results, in which *PSMA* genes are highly involved in BRCA and poor prognoses.

## 5. Conclusions

Collectively, he present study could provide useful bioinformatic evidence and potential target genes for prospective studies on the role of *PSMA* genes in BRCA disease.. Most of all, by integrating multiple high-throughput databases, our study revealed that *PSMA* genes have prognostic and predictive value in BRCA. Therefore, our results can be used as hints for the further examination of this family, and, possibly, they can serve as distinctive biomarkers and potential prognosticators in BRCA. Further research and attention to *PSMA* family genes will help us better understand BRCA progression and offer new insights into identifying the biomarkers or potential therapeutic targets of BRCA.

## Data Availability

The datasets used and/or analyzed during the current study are available from the corresponding author on reasonable request.

## Supplementary Materials

The following are available online at https://www.mdpi.com/article/10.3390/diagnostics11122220/s1, Supplementary Figure S1. Heatmap of DNA methylation expression levels of the *PSMA* gene family in breast cancer by MethSurv platform. cg07435350, cg26165081, cg26868250 of *PSMA1*; cg10778455, cg106226670, cg15202134 of *PSMA2*; cg08095532, cg14211735 of *PSMA4*; cg08250978, cg13170147 of *PSMA5*; cg01757308 of *PSMA6*; cg17665883 of *PSMA7*; cg11858305, cg15865827, cg00262344, cg06377543, cg03162994, cg22027766, cg259833544, cg01070760, cg21248196 of *PSMA8*; displays the highest level of DNA methylation in breast cancer. Table S1: Pathway analysis of proteasome 20S subunit alpha 1 (*PSMA1*)-coexpressed genes from public breast cancer databases using the MetaCore database (with *p* < 0.01 set as the cutoff value) Table S2: Pathway analysis of proteasome 20S subunit alpha 2 (*PSMA2*)-coexpressed genes from public breast cancer databases using the MetaCore database (with *p* < 0.01 set as the cutoff value). Table S3: Pathway analysis of proteasome 20S subunit alpha 3 (*PSMA3*)-coexpressed genes from public breast cancer databases using the MetaCore database (with *p* < 0.01 set as the cutoff value). Table S4: Pathway analysis of proteasome 20S subunit alpha 4 (*PSMA4*)-coexpressed genes from public breast cancer databases using the MetaCore database (with *p* < 0.01 set as the cutoff value). Table S5: Pathway analysis of proteasome 20S subunit alpha 5 (*PSMA5*)-coexpressed genes from public breast cancer databases using the MetaCore database (with *p* < 0.01 set as the cutoff value). Table S6: Pathway analysis of proteasome 20S subunit alpha 6 (*PSMA6*)-coexpressed genes from public breast cancer databases using the MetaCore database (with *p* < 0.01 set as the cutoff value). Table S7: Pathway analysis of proteasome 20S subunit alpha 7 (*PSMA7*)-coexpressed genes from public breast cancer databases using the MetaCore database (with *p* < 0.01 set as the cutoff value). Table S8: Pathway analysis of proteasome 20S subunit alpha 8 (*PSMA8*)-coexpressed genes from public breast cancer databases using the MetaCore database (with *p* < 0.01 set as the cutoff value).
