# Supplementary material for: Prognostic and Genomic Analysis of Proteasome 20S Subunit Alpha (PSMA) Family Members in Breast Cancer
Source: Diagnostics (Basel). 2021 Nov 27;11(12):2220. doi: 10.3390/diagnostics11122220 (PMC8699889; doi:10.3390/diagnostics11122220)
Supplement: Supplementary file 1 [file diagnostics-11-02220-s001.zip › diagnostics-1440615-supplementary.pdf]

Supplementary Material:

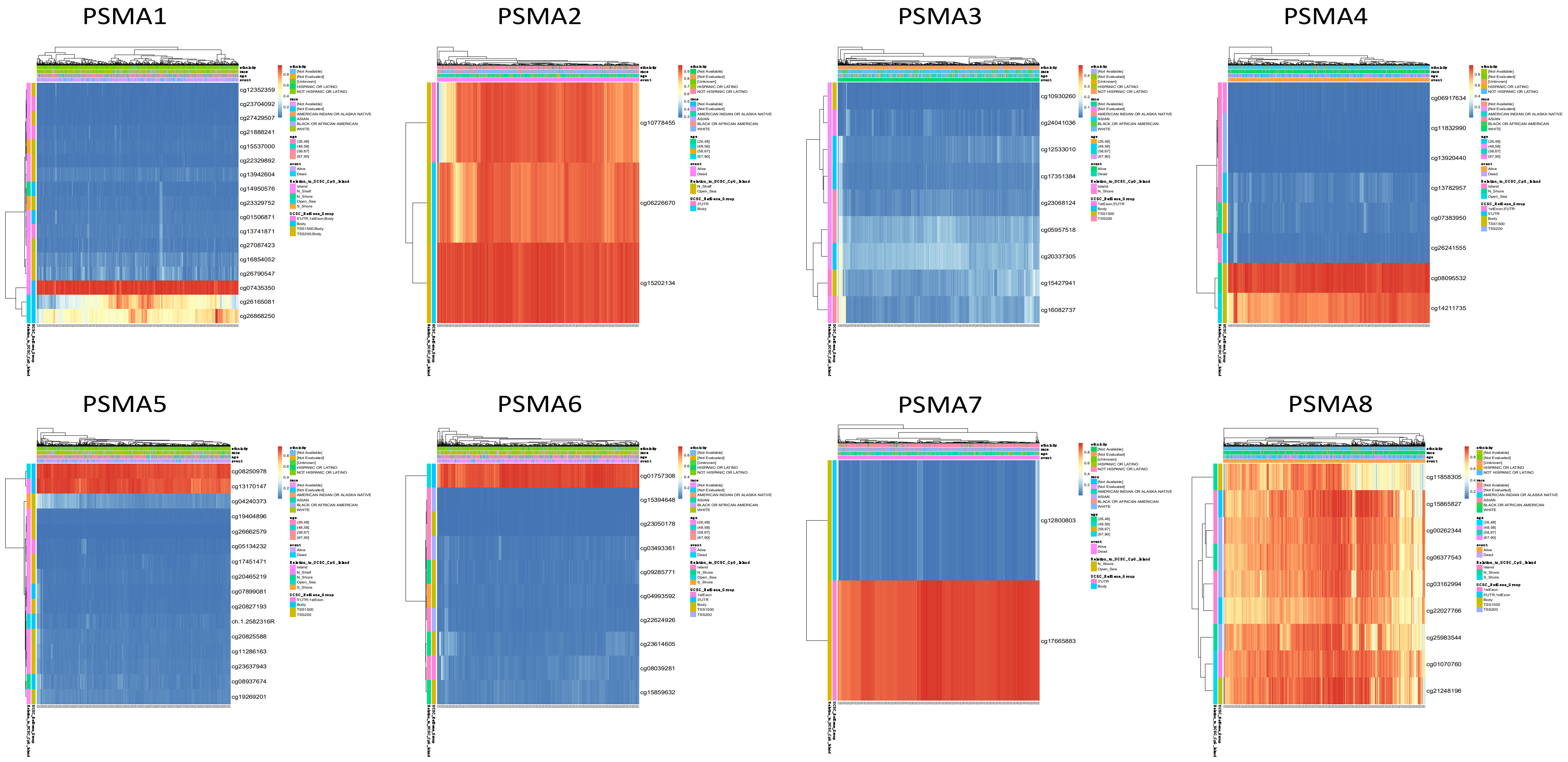

Supplementary Figure S1. Heatmap of DNA methylation expression levels of the PSMA gene family in breast cancer by MethSurv platform. cg07435350, cg26165081, cg26868250 of PSMA1; cg10778455,

cg106226670, cg15202134 of PSMA2; cg08095532, cg14211735 of PSMA4; cg08250978, cg13170147 of PSMA5; cg01757308 of PSMA6; cg17665883 of PSMA7; cg11858305, cg15865827, cg00262344, cg06377543, cg03162994, cg22027766, cg259833544, cg01070760, cg21248196 of PSMA8; displays the highest level of DNA methylation in breast cancer

Table S1: Pathway analysis of proteasome 20S subunit alpha 1 (PSMA1)-coexpressed genes from public breast cancer databases using the MetaCore database (with *p*<0.01 set as the cutoff value)

No.	Map	*p* Value	Network objects from active data
**1**	Cell cycle_Role of SCF complex in cell cycle regulation	6.42E-08	CDK4, Ubiquitin, Emi1, Skp2/TrCP/FBXW, Chk1, RING-box protein 1, Wee1, CKS1
**2**	Immune response_Antigen presentation by MHC class I, classical pathway	1.01E-06	PSMB5, PSMB9, HSP70, Calnexin, IDE, Beta-2-microglobulin, Sec24, Sec23, PSMB8(LMP7)
**3**	Apoptosis and survival_Regulation of apoptosis by mitochondrial proteins	1.20E-06	Cathepsin L, DNM1L (DRP1), SLC25A3, Cyclin A, Metaxin 1, VDAC 1, PP2C, LETM1, PARL, VDAC 2, SOD1, Mitofusin 1
**4**	Proteolysis_Putative ubiquitin pathway	4.36E-06	UBCH8, Ubiquitin, HSP70, FBXW7, RING-box protein 1, TRAF6
**5**	Ubiquinone metabolism	1.48E-05	NDUFB4, NDUFA4, NDUFB6, NDUFS4, NDUFB3, NDUFB2, coenzyme Q2 homolog, prenyltransferase (yeast), NDUFB5, DAP13
**6**	Oxidative stress_Role of Sirtuin1 and PGC1-alpha in activation of antioxidant defense system	2.25E-05	SCPX, PRDX3, Thioredoxin, PRDX1, TXNRD1, GCL reg, MT-TRX, SOD1
**7**	Cell cycle_Spindle assembly and chromosome separation	4.05E-05	Ubiquitin, Importin (karyopherin)-alpha, Importin (karyopherin)-beta, Tubulin alpha, MAD2a, Ran
**8**	Cell cycle_ESR1 regulation of G1/S transition	5.75E-05	CDK4, Ubiquitin, Cyclin A2, Cyclin A, Skp2/TrCP/FBXW, CKS1
**9**	NRF2 regulation of oxidative stress response	8.88E-05	Ubiquitin, MEK1(MAP2K1), Thioredoxin, PRDX1, TXNRD1, GCL reg, SOD1
**10**	Oxidative stress_Role of ASK1 under oxidative stress	8.88E-05	UNRIP, Thioredoxin, PRDX1, MT-TRX, TRAF6, SOD1, 14-3-3
**11**	Cigarette smoke-mediated regulation of NRF2-antioxidant pathway in airway epithelial cells	2.37E-04	PRDX1, TXNRD1, GCL reg, GSTO1, TALDO

**12**	DeltaF508-CFTR traffic / ER-to-Golgi in CF	2.94E-04	Ubiquitin, Sec24, Sec23, SAR1
**13**	wtCFTR traffic / ER-to-Golgi (normal)	2.94E-04	Ubiquitin, Sec24, Sec23, SAR1
**14**	Transport_RNA[?] regulation pathway	3.72E-04	NUP54, Importin (karyopherin)-alpha, Importin (karyopherin)-beta, Ran
**15**	Cell cycle_Role of APC in cell cycle regulation	3.83E-04	Geminin, Emi1, Cyclin A, MAD2a, CKS1
**16**	DNA damage_ATM/ATR regulation of G2/M checkpoint: cytoplasmic signaling	4.89E-04	JAB1, Chk1, PP1-cat, DCK, Nucleolysin TIAR, 14-3-3
**17**	Signal transduction_Adenosine A2B receptor signaling pathway	5.01E-04	Ubiquitin, LDHA, RAP-1A, JAB1, MEK1/2, APIP, G-protein alpha-q/11
**18**	Cell cycle_Chromosome condensation in prometaphase	6.93E-04	CAP-C, Cyclin A, CAP-G/G2, CAP-E
**19**	Ubiquitin-proteasome system in Huntington's disease	8.34E-04	PSMC2, Ubiquitin, PSMB9, HIP-2
**20**	Cell cycle_Regulation of G1/S transition (part 1)	8.68E-04	CDK4, Ubiquitin, Cyclin A, Skp2/TrCP/FBXW, RING-box protein 1
**21**	Regulation of degradation of deltaF508-CFTR in CF	9.79E-04	Ubiquitin, HSP70, RNF4, Hdj-2, HSC70
**22**	COVID-19: SARS-CoV-2 effects on infected tissues	1.04E-03	Cathepsin L, eIF3S1, Thioredoxin, Karyopherin alpha 2, MIP-1-beta, SRP54, 4EHP, STAT2, RAE1, TRAF6
**23**	Proteolysis_Role of Parkin in the Ubiquitin-Proteasomal Pathway	1.17E-03	UBCH8, HSP70, FBXW7, Tubulin alpha
**24**	CFTR folding and maturation (normal and CF)	1.17E-03	HSP70, Calnexin, Hdj-2, p23 co-chaperone
**25**	HSP70 and HSP40-dependent folding in Huntington's disease	1.38E-03	Ubiquitin, HSP70, Hdj-2, HSC70

Table S2: Pathway analysis of proteasome 20S subunit alpha 2 (PSMA2)-coexpressed genes from public breast cancer databases using the MetaCore database (with *p*<0.01 set as the cutoff value)

**No.**	**Map**	***p* Value**	**Network objects from active data**
1	Propionate metabolism p.2	2.08E-07	SUCLG1, ACAT1, HADHA, SCS-G, ACADM, SCS-A, Acetyl-CoA acyltransferase, SUCLG2, HADHB, HCDH
2	Role of XBP1 protein in multiple myeloma	2.56E-05	SERP1, PSMA5, DnaJB9, XBP1, PSMA6

**3**	Development_Positive regulation of WNT/Beta-catenin signaling in the cytoplasm	7.73E-05	GSKIP, UBE2B, BIG1, PP1-cat, USP47, PP2A catalytic, SIAH2, 14-3-3
**4**	Leucine, isoleucine and valine metabolism.p.2	1.02E-04	ACAT1, HADHA, AUMH, Acetyl-CoA acyltransferase, HADHB, HCD2, HCDH, MCEE
**5**	Tricarbonic acid cycle	1.26E-04	SUCLG1, SUCB1, SCS-G, DLDH, SCS-A, SUCLG2, SDHD
**6**	N-Glycan biosynthesis p1	3.58E-04	ALG10-A, ALG11, ALG5, DPM1 (cat), ALG6, OST48, ALG10B
**7**	Ubiquinone metabolism	4.24E-04	NDUFAB1, NDUFA4, NDUFA10, NDUFS4, NDUFB5, NDUFV2, NDUFA5
**8**	Signal transduction_Angiotensin II signaling via Beta-arrestin	6.18E-04	RhoA, Clathrin heavy chain, PP2A catalytic, eIF4E, p23 co-chaperone, 14-3-3
**9**	wtCFTR and deltaF508-CFTR traffic / Generic schema (normal and CF)	6.31E-04	Vps25, TSG101, Clathrin heavy chain, Rab-5A, Rab-11A
**10**	CREB1-dependent transcription deregulation in Huntington's Disease	1.24E-03	NDUFB5, ACADM, GRP75, SOD1
**11**	Cell cycle_Initiation of mitosis	1.24E-03	Cyclin H, CAK complex, MAT1, CDK7
**12**	Mitochondrial unsaturated fatty acid beta-oxidation	1.38E-03	HADHA, ACADM, Acetyl-CoA acyltransferase, HADHB, HCDH
**13**	Development_G-protein-mediated regulation of MAPK-ERK signaling	1.53E-03	RAP-1A, GMF, SynGAP, G-protein alpha-i family, Calmodulin
**14**	DNA damage_Nucleotide excision repair	1.83E-03	ERCC8, USP45, PCNA, E2N(UBC13), XPA, NEDD4
**15**	Cell cycle_Start of DNA replication in early S phase	2.73E-03	RPA3, ORC4L, ORC5L, PP2A catalytic
**16**	Regulation of AKT(PKB)/ GSK3 beta cascade in bipolar disorder	3.13E-03	ESR1 (membrane), 14-3-3 beta/alpha, G-protein alpha-i family, G-protein alpha-i3, Calmodulin
**17**	Oxidative stress_Role of ASK1 under oxidative stress	3.13E-03	ZPR9, UNRIP, PP2A catalytic, SOD1, 14-3-3
**18**	Transcription_Role of the non-genomic action of Retinoic acid and phosphorylation of Retinoic acid receptors in the initiation of transcription	3.80E-03	Cyclin H, CAK complex, MAT1, CDK7

**19**	Altered Ca2+ handling in heart failure	3.80E-03	PP1-cat, G-protein alpha-i family, PP2A catalytic, Calmodulin
**20**	Immune response_Lipoxins and Resolvin E1 inhibitory action on neutrophil functions	3.80E-03	NFKBIB, I-kB, G-protein alpha-i family, SOD1
**21**	Development_Negative regulation of WNT/Beta-catenin signaling in the cytoplasm	4.07E-03	KLHL12, WWP1, PEG3, PP1-cat, Casein kinase I alpha, PP2A catalytic
**22**	Propionate metabolism p.1	4.22E-03	3HIDH, HCD2, HCDH, MCEE
**23**	Peroxisomal branched chain fatty acid oxidation	4.58E-03	DHB4, HACL1, HADHA, Acetyl-CoA acyltransferase, HADHB, MCEE
**24**	Signal transduction_Cyclic AMP signaling	5.13E-03	KDELR, RAP-1A, G-protein alpha-i family, Calmodulin
**25**	G-protein signaling_Rap1A regulation pathway	6.18E-03	RAP-1A, p120GAP, G-protein alpha-i family, NEDD4

Table S3: Pathway analysis of proteasome 20S subunit alpha 3 (PSMA3)-coexpressed genes from public breast cancer databases using the MetaCore database (with *p*<0.01 set as the cutoff value)

**No.**	**Map**	***p* Value**	**Network objects from active data**
**1**	Immune response_Antigen presentation by MHC class I, classical pathway	1.44E-14	PSMB1, PSMB5, MHC Class I alpha chain, PSME2, MHC class I, PSMB9, HSP70, IDE, Beta-2-microglobulin, Sec24, PA28 (11S regulator), Impas 1, Sec23, PSMB2, PSME1, PSMB8(LMP7)
**2**	Cell cycle_Spindle assembly and chromosome separation	2.18E-08	Importin (karyopherin)-alpha, CSE1L, Aurora-A, KNSL1, HEC, Tubulin alpha, Cyclin B, MAD2a, Securin
**3**	Immune response_Induction of the antigen presentation machinery by IFN-gamma	1.72E-06	PSME2, MHC class I, PSMB9, Beta-2-microglobulin, HLAC, HLA-DRA1, HLAB, PSME1, PSMB8(LMP7)
**4**	Cell cycle_Role of APC in cell cycle regulation	4.28E-06	BUB1, Geminin, Cyclin A, Aurora-A, Cyclin B, MAD2a, Securin
**5**	Immune response_Antigen presentation by MHC class I: cross-presentation	9.07E-06	LLIR, Cathepsin L, MHC class I, HSP70, Dectin-1, Rab8B, HSP105, Fc epsilon RI gamma, IP-30, MSR1, RanBPM

**6**	CFTR folding and maturation (normal and CF)	9.28E-06	HSP70, Aha1, HSP105, Hdj-2, FKBP8, p23 co-chaperone
**7**	Cell cycle_The metaphase checkpoint	9.86E-06	BUB1, SPBC25, Aurora-A, HEC, CDCA1, HZwint-1, MAD2a
**8**	DNA damage_ATM/ATR regulation of G2/M checkpoint: cytoplasmic signaling	1.21E-05	Cyclin B1, JAB1, PP2A regulatory, Chk1, Aurora-A, DCK, Nucleolysin TIAR, B56G
**9**	Immune response_Antigen presentation by MHC class II	4.80E-05	MHC class II alpha chain, LLIR, Cathepsin L, Dectin-1, Fc epsilon RI gamma, IP-30, CDC42, Cathepsin V, MARCH1, HSC70, SPPL2a
**10**	Cell cycle_Role of Nek in cell cycle regulation	5.41E-05	Cyclin B1, Aurora-A, HEC, Tubulin alpha, MAD2a, Histone H1
**11**	Cell cycle_Chromosome condensation in prometaphase	6.92E-05	Cyclin A, Aurora-A, CAP-E, Cyclin B, Histone H1
**12**	Putative pathways of MHC class I-dependent postsynaptic long-term depression in major depressive disorder	6.92E-05	MHC Class I alpha chain, MHC class I, Beta-2-microglobulin, PA28 (11S regulator), HLAB
**13**	COVID-19: SARS-CoV-2 effects on infected tissues	1.34E-04	Cathepsin L, eIF3S1, MDA-5, Thioredoxin, Karyopherin alpha 2, MIP-1-beta, I-TAC, SRP54, CCL8, Karyopherin alpha 1, IP10, SRP19
**14**	Immune response_Immunological synapse formation	1.69E-04	MHC class I, Cytohesin1, CDC42, Fyn, CD80, WaspIP, NCK1
**15**	Regulation of degradation of deltaF508-CFTR in CF	1.71E-04	HSP70, Aha1, HSP105, Derlin1, Hdj-2, HSC70
**16**	Cell cycle_Nucleocytoplasmic transport of CDK/Cyclins	1.79E-04	CDK4, Importin (karyopherin)-alpha, Cyclin B1, Cyclin A
**17**	Cell cycle_Initiation of mitosis	2.05E-04	Cyclin B1, KNSL1, Cyclin B2, MAT1, Histone H1
**18**	DNA damage_ATM/ATR regulation of G1/S checkpoint	3.39E-04	CDK4, PCNA, PP2A regulatory, Cyclin A, Chk1, B56G
**19**	Cigarette smoke-mediated regulation of NRF2-antioxidant pathway in airway epithelial cells	3.50E-04	PRDX1, TXNRD1, GCL reg, GSTO1, ME1
**20**	Abnormalities in cell cycle in SCLC	3.50E-04	CDK4, PCNA, Cyclin B1, Cyclin A, Max
**21**	Immune response_IFN-alpha/beta signaling via JAK/STAT	3.60E-04	MHC class I, HIF1A, I-TAC, GBP1, RSAD2, IFI27, IP10
**22**	Cell cycle_Start of DNA replication in early S phase	5.64E-04	RPA3, Geminin, MCM10, ORC6L, Histone H1
**23**	Role of tumor-infiltrating B cells in anti-tumor immunity	7.68E-04	MHC class I, IRF4, KTN1, CD38, I-TAC, DHFR, CXCR4, IP10
**24**	Immune response_IFN-alpha/beta signaling via PI3K and NF-	9.52E-04	CDK4, PCNA, I-TAC, Cyclin A, GBP1, DHFR, RSAD2, NMI

	kB pathways		
**25**	NRF2 regulation of oxidative stress response	1.04E-03	Thioredoxin, PRDX1, TXNRD1, Fyn, GCL reg, SOD1

Table S4: Pathway analysis of proteasome 20S subunit alpha 4 (PSMA4)-coexpressed genes from public breast cancer databases using the MetaCore database (with *p*<0.01 set as the cutoff value)

No.	Map	*p* Value	Network objects from active data
**1**	Immune response_IFN-alpha/beta signaling via JAK/STAT	2.93E-11	MHC class I, CCL2, TAP1 (PSF1), HIF1A, I-TAC, MIG, GBP1, ISG54, RSAD2, ISG20, IFI27, IFN-gamma, STAT1, IP10, GBP4
**2**	Cell cycle_Role of APC in cell cycle regulation	2.04E-10	Nek2A, BUB1, Tome-1, Geminin, Emi1, Cyclin A, Aurora-A, Cyclin B, MAD2a, Securin, CKS1
**3**	Cell cycle_Spindle assembly and chromosome separation	2.99E-10	Nek2A, Importin (karyopherin)-alpha, CSE1L, Aurora-A, KNSL1, HEC, Tubulin alpha, Cyclin B, MAD2a, Securin, Tubulin (in microtubules)
**4**	Immune response_Antigen presentation by MHC class I, classical pathway	6.71E-10	PSMB1, PSMB5, MHC Class I alpha chain, PSME2, MHC class I, PSMB9, HSP70, TAP1 (PSF1), IDE, Beta-2-microglobulin, PSMB2, IFN-gamma, PSMB8(LMP7)
**5**	Cell cycle_The metaphase checkpoint	8.68E-10	Nek2A, BUB1, SPBC25, CENP-A, Aurora-A, HEC, CDCA1, HZwint-1, MAD2a, CENP-E, AF15q14
**6**	Release of pro-inflammatory mediators and elastolytic enzymes by alveolar macrophages in COPD	3.31E-07	MMP-12, Cathepsin L, CCL2, MMP-1, IFN-gamma, STAT1, IP10, HDAC2
**7**	Immune response_Induction of the antigen presentation machinery by IFN-gamma	7.58E-07	PSME2, MHC class I, PSMB9, TAP1 (PSF1), Beta-2-microglobulin, HLA-E, HLA-F, IFN-gamma, PSMB8(LMP7), STAT1
**8**	Cell cycle_Role of Nek in cell cycle regulation	1.03E-06	Nek2A, Tubulin beta, Cyclin B1, Aurora-A, HEC, Tubulin alpha, MAD2a, Tubulin (in microtubules)
**9**	COVID-19: immune dysregulation	1.5E-06	CCL5, MHC class I, MDA-5, CCL2, HIF1A, MIP-1-beta, MIG, CCL8, HLA-E,

			Granzyme B, IFN-gamma, sIL2RA, IP10
**10**	Glomerular injury in Lupus Nephritis	3.67E-06	ATF-4, CCL5, MDA-5, CCL2, HIF1A, MEK1/2, MMP-1, Annexin II, VCAM1, IFN-gamma, STAT1, IP10
**11**	Signal transduction_PDGF signaling via JAK-STAT and reactive oxygen species (ROS)	6.11E-06	CCL5, SOD2, CCL2, 14-3-3 gamma, HIF1A, I-TAC, MIG, STAT1, IP10, CCL13
**12**	Role of Bregs in attenuation of T and NK cells mediated anti-tumor immune responses	7.62E-06	MIG, CTLA-4, CD80, IL-2R alpha chain, Granzyme B, Lck, IFN-gamma, IP10
**13**	Oxidative stress_Role of ASK1 under oxidative stress	8.02E-06	HPK38, SOD2, 14-3-3 gamma, Thioredoxin, PRDX1, MT-TRX, Glutaredoxin, SOD1, 14-3-3
**14**	Inter-cellular relations in COPD (general schema)	8.17E-06	CCL2, I-TAC, MIG, Granzyme B, IFN-gamma, IP10, HDAC2
**15**	Immune response_CD16 signaling in NK cells	9.21E-06	PLA2, MEK1(MAP2K1), CDC42, Calcineurin B (regulatory), HLA-E, CD94, Lck, IFN-gamma, Calmodulin, WaspIP
**16**	Chemotaxis_CXCR3-A signaling	9.21E-06	MEK1/2, I-TAC, MIG, CDC42, Granzyme B, Lck, IFN-gamma, Calmodulin, STAT1, IP10
**17**	Cell cycle_Nucleocytoplasmic transport of CDK/Cyclins	1.75E-05	Importin (karyopherin)-alpha, Cyclin B1, Cyclin A, CRM1, Cyclin D
**18**	Role of tumor-infiltrating B cells in anti-tumor immunity	1.95E-05	MHC class I, IRF4, CD38, I-TAC, MIG, DHFR, Granzyme B, IFN-gamma, STAT1, IP10, CXCL13
**19**	Microsatellite instability in colorectal cancer	1.98E-05	PCNA, MHC class I, MutSalpha complex, Beta-2-microglobulin, MSH6, EXO1, MSH2
**20**	Common mechanisms of Th17 cell migration	2.58E-05	CCL5, CCL2, MIP-1-beta, I-TAC, MIG, CCL8, VCAM1, IP10
**21**	Breakdown of CD4+ T cell peripheral tolerance in type 1 diabetes mellitus	3.01E-05	ICOS, Ku70, CTLA-4, CD80, IL-2R alpha chain, Lck, PTPN22, IFN-gamma
**22**	T cell generation in COPD	3.12E-05	MHC class I, CCL2, CTLA-4, CCL8, CD80, CCL13
**23**	Rheumatoid arthritis (general schema)	3.51E-05	MMP-1, CD80, IL-2R alpha chain, VCAM1, CD2, PTPN22, IFN-gamma,

			BAFF(TNFSF13B)
**24**	Attenuation of IFN type I signaling in melanoma cells	3.54E-05	MHC class I, PSMB9, TAP1 (PSF1), I-TAC, PSMB8(LMP7), STAT1, IP10
**25**	Cooperative action of IFN-gamma and TNF-alpha on astrocytes in multiple sclerosis	5.07E-05	MHC class I, CCL2, HLA-E, CD80, IFN-gamma, STAT1, IP10

Table S5: Pathway analysis of proteasome 20S subunit alpha 5 (PSMA5)-coexpressed genes from public breast cancer databases using the MetaCore database (with *p*<0.01 set as the cutoff value)

**No.**	**Map**	***p* Value**	**Network objects from active data**
**1**	Cell cycle_The metaphase checkpoint	4.68E-13	Nek2A, BUB1, SPBC25, CENP-A, Aurora-A, Aurora-B, HEC, CDCA1, HZwint-1, MAD2a, Survivin, CENP-H, CENP-E, AF15q14
**2**	Immune response_IFN-alpha/beta signaling via JAK/STAT	1.06E-12	IRF1, MHC class I, RAP-1A, TAP1 (PSF1), OAS1, IFI6, I-TAC, MIG, GBP1, RSAD2, ISG20, IFI27, IFN-gamma, STAT1, IP10, GBP4, ISG15
**3**	Immune response_Antigen presentation by MHC class I, classical pathway	1.31E-12	PSMB1, PSMB5, MHC Class I alpha chain, PSME2, MHC class I, PSMB9, HSP70, TAP1 (PSF1), IDE, Beta-2-microglobulin, TAP, Sec24, PSMB2, IFN-gamma, PSMB8(LMP7), TAP2 (PSF2)
**4**	Cell cycle_Role of APC in cell cycle regulation	1.66E-12	Nek2A, BUB1, Tome-1, Geminin, Emi1, Cyclin A, Aurora-A, Aurora-B, Cyclin B, MAD2a, Securin, ORC1L, CKS1
**5**	Cell cycle_Spindle assembly and chromosome separation	2.67E-12	Nek2A, Importin (karyopherin)-alpha, TPX2, CSE1L, Aurora-A, KNSL1, Aurora-B, HEC, Tubulin alpha, Cyclin B, MAD2a, Securin, Ran
**6**	Attenuation of IFN type I signaling in melanoma cells	6.20E-08	IRF1, MHC class I, PSMB9, TAP1 (PSF1), I-TAC, TAP, PSMB8(LMP7), TAP2 (PSF2), STAT1, IP10
**7**	Putative pathways of MHC class I-dependent postsynaptic long-term depression in major depressive disorder	6.57E-08	MHC Class I alpha chain, MHC class I, TAP1 (PSF1), Beta-2-microglobulin, TAP, CD3 zeta, IFN-gamma, TAP2 (PSF2)

**8**	Role of Bregs in attenuation of T and NK cells mediated anti-tumor immune responses	1.80E-07	CD3, MIG, CTLA-4, CD80, IL-2R alpha chain, CD3 zeta, Granzyme B, FasL(TNFSF6), IFN-gamma, IP10
**9**	Immune response_Induction of the antigen presentation machinery by IFN-gamma	2.58E-07	IRF1, PSME2, MHC class I, PSMB9, TAP1 (PSF1), Beta-2-microglobulin, HLA-F, IFN-gamma, PSMB8(LMP7), TAP2 (PSF2), STAT1
**10**	Immune response_IFN-gamma actions on extracellular matrix and cell differentiation	3.15E-07	IRF1, PSME2, GCH1, OAS1, EZH2, OAS2, IFN-gamma, 2'-5'-oligoadenylate synthetase, STAT1, IP10, HDAC2
**11**	Immune response_Induction of apoptosis and inhibition of proliferation mediated by IFN-gamma	7.13E-07	IRF1, RAP-1A, Caspase-1, OAS1, IDO1, GBP1, FasL(TNFSF6), Caspase-3, IFN-gamma, STAT1
**12**	Role of tumor-infiltrating B cells in anti-tumor immunity	1.97E-06	MHC class I, CD38, I-TAC, MIG, DHFR, G-protein alpha-i family, Granzyme B, FasL(TNFSF6), CXCR4, IFN-gamma, STAT1, IP10, CXCL13
**13**	Cell cycle_Role of Nek in cell cycle regulation	2.57E-06	Nek2A, Cyclin B1, TPX2, Aurora-A, HEC, Tubulin alpha, MAD2a, Ran
**14**	CD8+ Tc1 cells in allergic contact dermatitis	6.48E-06	MHC class I, Caspase-1, CD80, Granzyme B, FasL(TNFSF6), Caspase-3, IFN-gamma
**15**	iNKT cell-keratinocyte interactions in allergic contact dermatitis	6.68E-06	IRF1, MHC class I, CD3, Beta-2-microglobulin, FasL(TNFSF6), IFN-gamma, STAT1, IP10
**16**	DNA damage_G2 checkpoint in response to DNA mismatches	1.81E-05	PCNA, MutSalpha complex, Chk2, MSH6, Chk1, EXO1, MSH2
**17**	Cell cycle_Chromosome condensation in prometaphase	2.09E-05	Cyclin A, CAP-G/G2, Aurora-A, Aurora-B, CAP-E, Cyclin B
**18**	Cell cycle_Start of DNA replication in early S phase	2.85E-05	RPA3, Geminin, MCM10, ORC6L, CDC7, ORC1L, CDC45L
**19**	COVID-19: immune dysregulation	3.00E-05	IRF1, MHC class I, MDA-5, Caspase-1, CD3, MIG, CCL8, Granzyme B, IFN-gamma, sIL2RA, IP10, ISG15
**20**	Cell cycle_Nucleocytoplasmic transport of CDK/Cyclins	3.16E-05	Importin (karyopherin)-alpha, Cyclin B1, Cyclin A, CRM1, Cyclin D
**21**	NK cells in allergic contact dermatitis	4.34E-05	IRF1, MIG, CCL20, FasL(TNFSF6), IFN-gamma, STAT1, IP10
**22**	Microsatellite instability in colorectal cancer	4.34E-05	PCNA, MHC class I, MutSalpha complex, Beta-2-microglobulin, MSH6, EXO1, MSH2

23	Immune escape mechanisms in Prostate Cancer	6.19E-05	MHC class I, TAP1 (PSF1), Beta-2-microglobulin, TAP, IFN-gamma, TAP2 (PSF2)
24	T cell generation in COPD	6.19E-05	MHC class I, CTLA-4, CCL8, CD80, CCL20, CCL13
25	Breakdown of CD4+ T cell peripheral tolerance in type 1 diabetes mellitus	7.18E-05	ICOS, CD3, CTLA-4, CD80, IL-2R alpha chain, PTPN22, IFN-gamma, PTPN2

Table S6: Pathway analysis of proteasome 20S subunit alpha 6 (PSMA6)-coexpressed genes from public breast cancer databases using the MetaCore database (with *p*<0.01 set as the cutoff value)

No.	Map	*p* Value	Network objects from active data
1	Immune response_Antigen presentation by MHC class I, classical pathway	4.32E-16	PSMB5, MHC Class I alpha chain, PSME2, MHC class I, PSMB9, HSP70, Calnexin, IDE, Beta-2-microglobulin, HSP90 alpha, Sec24, PA28 (11S regulator), Impas 1, Sec23, PSMB2, PSME1, PSMB8(LMP7)
2	Apoptosis and survival_Regulation of apoptosis by mitochondrial proteins	5.48E-09	Cathepsin L, p38alpha (MAPK14), SLC25A3, Cyclin A, Smac/Diablo, VDAC 1, JNK(MAPK8-10), p38 MAPK, JNK2(MAPK9), PP2C, PARL, MPTP complex, VDAC 2, SOD1, Mitofusin 1
3	Possible regulation of HSF-1/ chaperone pathway in Huntington's disease	1.52E-07	HSP90, PLA2, HSP70, HSP90 alpha, SUMO-2, JNK(MAPK8-10), p23 co-chaperone
4	Cigarette smoke-induced oxidative stress and apoptosis in airway epithelial cells	4.26E-07	PRDX3, Thioredoxin, PRDX1, VDAC 1, JNK(MAPK8-10), p38 MAPK, JNK2(MAPK9), GRP78, Glutaredoxin, SOD1
5	CFTR folding and maturation (normal and CF)	4.28E-07	HSP70, Calnexin, HSP105, HSP90 alpha, Hdj-2, FKBP8, p23 co-chaperone
6	Regulation of degradation of deltaF508-CFTR in CF	1.20E-06	HSP90, Ubiquitin, HSP70, HSP105, SUMO-2, Derlin1, Hdj-2, HSC70
7	Immune response_Induction of the antigen presentation machinery by IFN-gamma	1.38E-06	PSME2, MHC class I, PSMB9, Beta-2-microglobulin, HLAC, PRMT5, HLAB, PSME1, PSMB8(LMP7)

**8**	Oxidative stress_Role of ASK1 under oxidative stress	1.63E-06	Thioredoxin, PRDX1, JNK(MAPK8-10), p38 MAPK, MT-TRX, JNK2(MAPK9), Glutaredoxin, SOD1, 14-3-3
**9**	DNA damage_Nucleotide excision repair	1.86E-06	ERCC8, ERCC6, PCNA, HMG14, Histone H2A, E2N(UBC13), Histone H2B, NEDD8, Histone H4, Histone H3
**10**	Immune response_Antigen presentation by MHC class II	6.87E-06	HSP90, Cathepsin L, 14-3-3 beta/alpha, HSP90 alpha, CDC42, Cathepsin V, JNK(MAPK8-10), p38 MAPK, Legumain, MARCH1, HSC70, SPPL2a
**11**	DNA damage_ATM/ATR regulation of G2/M checkpoint: cytoplasmic signaling	1.00E-05	JAB1, p38alpha (MAPK14), p38 MAPK, JNK2(MAPK9), DCK, Nucleolysin TIAR, Histone H3, 14-3-3
**12**	HSP70 and HSP40-dependent folding in Huntington's disease	1.03E-05	HSP90, Ubiquitin, HSP70, HSP90 alpha, Hdj-2, HSC70
**13**	DNA damage_ATM activation by DNA damage	1.67E-05	HSP90, RCAD, HMG14, SOSSC, E2N(UBC13), Histone H2B, DMAP1, Histone H4, Histone H3
**14**	LRRK2 in neuronal apoptosis in Parkinson's disease	1.99E-05	PRDX3, Thioredoxin, VDAC 1, ANT, MPTP complex
**15**	dCTP/dUTP metabolism	2.35E-05	RRM2B, NDPK A, RRM2, POLG cat, Small RR subunit, NT5C3, DCK, POLE4, POLE2
**16**	Ubiquinone metabolism	2.35E-05	NDUFAB1, NDUFB6, NDUFS4, NDUFB3, NDUFB2, coenzyme Q2 homolog, prenyltransferase (yeast), NDUFB5, NDUFC1, DAP13
**17**	Regulation of degradation of wtCFTR	4.74E-05	HSP90, Ubiquitin, HSP105, Derlin1, HSC70
**18**	Cell cycle_Spindle assembly and chromosome separation	5.61E-05	Ubiquitin, Importin (karyopherin)-alpha, CSE1L, Tubulin alpha, MAD2a, Ran
**19**	LRRK2 in neurons in Parkinson's disease	5.61E-05	HSP90, Ubiquitin, MEK1/2, NSF, Clathrin heavy chain, 14-3-3
**20**	Putative pathways of MHC class I-dependent postsynaptic long-term depression in major depressive disorder	6.11E-05	MHC Class I alpha chain, MHC class I, Beta-2-microglobulin, PA28 (11S regulator), HLAB
**21**	Ubiquitin-proteasome system in Huntington's disease	7.77E-05	PSMC2, Ubiquitin, PSMB9, HIP-2, PA28 (11S regulator)
**22**	Transcription_Role of the non-genomic action of Retinoic acid and phosphorylation of Retinoic acid receptors in the initiation of	7.94E-05	Cyclin H, p38alpha (MAPK14), G-protein alpha-q, p38 MAPK, MAT1, Histone H3

	transcription		
23	Proteolysis_Putative ubiquitin pathway	9.76E-05	UBCH8, Ubiquitin, HSP70, E2N(UBC13), RING-box protein 1
24	Signal transduction_CXCR4 signaling via MAPKs cascades	1.13E-04	Ubiquitin, MEK1(MAP2K1), MEK1/2, JNK(MAPK8-10), p38 MAPK, CXCR4, G-protein alpha-13
25	Immune response_HSP60 and HSP70/ TLR signaling pathway	1.28E-04	Ubiquitin, MHC class I, HSP70, MEK1/2, JNK(MAPK8-10), p38 MAPK, E2N(UBC13)

Table S7: Pathway analysis of proteasome 20S subunit alpha 7 (PSMA7)-coexpressed genes from public breast cancer databases using the MetaCore database (with *p*<0.01 set as the cutoff value)

No.	Map	*p* Value	Network objects from active data
1	Cell cycle_Role of APC in cell cycle regulation	4.90E-17	BUB1, CDH1, Tome-1, Emi1, Cyclin A, Aurora-A, PLK1, Aurora-B, CDC25A, CDC20, SKP2, Cyclin B, MAD2a, Securin, ORC1L, CKS1
2	Cell cycle_The metaphase checkpoint	1.47E-14	INCENP, BUB1, SPBC25, CENP-A, Rod, Aurora-A, PLK1, Aurora-B, CDC20, HZwint-1, CENP-F, MAD2a, Survivin, CENP-E, AF15q14
3	Cell cycle_Spindle assembly and chromosome separation	2.09E-12	Importin (karyopherin)-alpha, TPX2, CSE1L, Aurora-A, KNSL1, Aurora-B, CDC20, Tubulin alpha, Cyclin B, MAD2a, Separase, Securin, Tubulin (in microtubules)
4	DNA damage_ATM/ATR regulation of G2/M checkpoint: nuclear signaling	1.25E-11	CDC25C, WDHD1, CDH1, HSF1, Histone H2AX, Chk2, Cyclin A, Chk1, PLK1, Cyclin B, Cyclin B2, TTK, DNA-PK, MDM2
5	DNA damage_Intra S-phase checkpoint	2.78E-11	BLM, CDH1, FANCD2, Histone H2AX, Chk2, MCM4, MCM3, Cyclin A, Chk1, CDC25A, MCM7, MCM10, DNA-PK, MCM2, Histone H3, MCM5, CDC45L
6	Cell cycle_Cell cycle (generic schema)	7.84E-11	CDC25C, CDK4, p107, Cyclin A, Cyclin E, CDC25A, E2F3, Cyclin B, E2F2, CDC25B
7	Cell cycle_Chromosome condensation in prometaphase	7.84E-11	INCENP, Cyclin A, CNAP1, CAP-G/G2, Aurora-A, CAP-D2/D3, Aurora-B, Cyclin B, TOP2, Histone H3

**8**	Abnormalities in cell cycle in SCLC	1.77E-10	CDK4, Cyclin A, Cyclin E, Aurora-B, SKP2, E2F3, E2F2, Histone H3, MDM2, Cyclin E2, CKS1
**9**	Cell cycle_Start of DNA replication in early S phase	1.09E-08	MCM4, MCM3, Cyclin E, MCM10, ORC6L, MCM4/6/7 complex, MCM2, ORC1L, MCM5, CDC45L
**10**	DNA damage_ATM/ATR regulation of G2/M checkpoint: cytoplasmic signaling	1.30E-08	CDC25C, UBE2C, BORA, Chk2, Chk1, Aurora-A, PLK1, Aurora-B, CDC25A, CDC25B, Histone H3, 14-3-3
**11**	Cell cycle_Initiation of mitosis	2.17E-08	CDC25C, Lamin B, PLK1, KNSL1, Cyclin B2, CDC25B, FOXM1, Kinase MYT1, Histone H3
**12**	Cell cycle_Transition and termination of DNA replication	2.17E-08	TOP2 alpha, Ribonuclease H1, Cyclin A, POLD reg (p68), MCM2, TOP2, FEN1, POLD cat (p125), DNA ligase I
**13**	Cell cycle_Role of SCF complex in cell cycle regulation	6.44E-08	CDK4, Emi1, Cyclin E, Skp2/TrCP/FBXW, Chk1, PLK1, CDC25A, SKP2, CKS1
**14**	Transcription_Ligand-dependent activation of the ESR1/SP pathway	8.98E-08	NCOA3 (pCIP/SRC3), TYSY, C/EBPbeta, VEGF-A, Cyclin E, CDC25A, NCOA1 (SRC1), Cyclin E2, ADA
**15**	Cell cycle_ESR1 regulation of G1/S transition	3.91E-07	CDK4, Cyclin A2, NCOA3 (pCIP/SRC3), Cyclin A, Cyclin E, Skp2/TrCP/FBXW, CDC25A, SKP2, CKS1
**16**	Transcription_Negative regulation of HIF1A function	4.57E-07	HSP90, HSP70, HIF1A, MCM3, Casein kinase I delta, HIF-prolyl hydroxylase, MCM7, Elongin C, MCM2, EGLN1, MDM2, MCM5
**17**	Reproduction_Progesterone-mediated oocyte maturation	1.34E-06	CDC25C, BUB1, c-Src, Aurora-A, PLK1, G-protein alpha-i family, CDC20, CDC25B, Kinase MYT1
**18**	Apoptosis and survival_DNA-damage-induced apoptosis	2.00E-06	BLM, FANCD2, Histone H2AX, Chk2, Chk1, DNA-PK
**19**	DNA damage_ATM/ATR regulation of G1/S checkpoint	3.13E-06	CDK4, Histone H2AX, Chk2, Cyclin A, Cyclin E, Chk1, CDC25A, MDM2, RFWD3
**20**	Higher ESR1 / ESR2 ratio in breast cancer	5.79E-06	Cyclin A2, NCOA2 (GRIP1/TIF2), HIF1A, VEGF-A, Cyclin E, CDC25A, SKP2, FOXM1
**21**	Cell cycle_Role of 14-3-3 proteins in cell cycle regulation	2.52E-05	CDC25C, Chk2, Chk1, CDC25A, 14-3-3 zeta/delta, CDC25B
**22**	Cell cycle_Role of Nek in cell cycle regulation	2.52E-05	Tubulin beta, TPX2, Aurora-A, Tubulin alpha, MAD2a, Histone H3, Tubulin (in

			microtubules)
23	Putative role of Estrogen receptor and Androgen receptor signaling in progression of lung cancer	3.32E-05	NCOA3 (pCIP/SRC3), E-cadherin, NCOA2 (GRIP1/TIF2), c-Src, G-protein alpha-i family, NCOA1 (SRC1), Caspase-3, RHEB2, 14-3-3
24	Immune response_IL-4-induced regulators of cell growth, survival, differentiation and metabolism	5.72E-05	CDK4, MCM6, MCM4, Cyclin A, Cyclin E, Cathepsin V, CDC25A, MCM5, SOCS1
25	Immune response_IFN-alpha/beta signaling via PI3K and NF-kB pathways	6.81E-05	CDK4, 4E-BP1, p107, Cyclin A, Cyclin E, GBP1, p19, DHFR, CDC25A, eIF4A, ISG15

Table S8: Pathway analysis of proteasome 20S subunit alpha 8 (PSMA8)-coexpressed genes from public breast cancer databases using the MetaCore database (with *p*<0.01 set as the cutoff value)

No.	Map	*p* Value	Network objects from active data
1	Immune response_NF-AT in immune response	2.99E-11	I-kB, ITK, Syk, CD28, Lyn, ZAP70, CD80, LAT, TRIM, NF-AT
2	Immune response_Inhibitory PD-1 signaling in T cells	4.49E-11	Csk, Syk, CD4, PD-1, CD28, Eomesodermin, PD-L1, Skp2/TrCP/FBXW, ZAP70, CD80
3	Breakdown of CD4+ T cell peripheral tolerance in type 1 diabetes mellitus	5.65E-10	IL-12 beta, CD4, PD-1, CD28, PD-L1, ZAP70, CD80, LAT, NF-AT
4	Immunological synapse between dendritic and CD8+ T cells in allergic contact dermatitis	1.38E-08	CD2, PD-1, CD137(TNFRSF9), CD28, PD-L1, CD80, CD137 ligand(TNFSF9)
5	B cell signaling in hematological malignancies	3.57E-08	I-kB, Syk, Cyclin D2, CD20, Lyn, ZAP70, CARD11, Semaphorin 4D, CD19
6	Immune response_T cell co-signaling receptors, schema	3.67E-08	CD2, PD-1, LAG3, CD137(TNFRSF9), CD28, PD-L1, CD80, CD137 ligand(TNFSF9)
7	Differences between Langerhans cells and dermal dendritic cells in allergic contact dermatitis	3.72E-08	CD4, CCR7, PD-1, CD28, PD-L1, CD80

**8**	Down-regulation of mast cell functions through ITIM-containing inhibitory receptors in asthma	4.06E-08	Csk, Syk, OX-2 receptor 1, Lyn, KLRG1, Semaphorin 4D, Fc epsilon RI beta
**9**	Immune response_Th1 and Th2 cell differentiation	7.18E-08	IL-12 beta, STAT1, CD4, CD137(TNFRSF9), CD28, CD80, CD137 ligand(TNFSF9)
**10**	T follicular helper cell dysfunction in SLE	1.41E-07	I-kB, CD84, STAT1, CD4, PD-1, CD28, CD80, CARD11, TRIM
**11**	Immune response_TCR alpha/beta signaling pathway	2.71E-07	I-kB, Csk, ITK, CD4, CD28, ZAP70, CD80, CARD11, LAT
**12**	SLE genetic marker-specific pathways in T cells	3.84E-07	NF-AT4(NFATC3), Aiolos, I-kB, Csk, IL-12 beta, MHC class II beta chain, PD-1, ZAP70, LAT
**13**	Immune response_CD28 signaling	6.96E-07	I-kB, ITK, CD28, ZAP70, CD80, LAT, NF-AT
**14**	Aberrant production of IL-2 and IL-17 in SLE T cells	1.01E-06	I-kB, Syk, CD4, CD229, ZAP70, LAT, CD3 epsilon
**15**	Immune response_Differentiation and clonal expansion of CD8+ T cells	1.43E-06	STAT1, CD4, CD137(TNFRSF9), CD28, CD80, CD137 ligand(TNFSF9)
**16**	Tumor metabolic pathways promoting development of Treg cells	2.91E-06	I-kB, CD4, PD-1, LAG3, CD28, PD-L1, CD80, CCL17
**17**	Modulation of tumor response to cytotoxic T cells by hypoxia in tumors	6.41E-06	PD-1, CD137(TNFRSF9), CD28, PD-L1, CD80, CD137 ligand(TNFSF9)
**18**	Immune response_Immunological synapse formation	1.13E-05	ITK, STK4, CD28, ZAP70, CD80, LAT
**19**	Role of IFN-beta in inhibition of Th1 cell differentiation in multiple sclerosis	1.42E-05	IL-12 beta, PD-1, CD28, PD-L1, CD80
**20**	Immune response_Fc epsilon RI pathway: signaling through Fyn and PI3K	2.06E-05	I-kB, Csk, Syk, Lyn, NF-AT, Fc epsilon RI beta
**21**	Immune response_Regulation of T cell function by CTLA-4	2.18E-05	CD28, Lyn, ZAP70, CD80, LAT
**22**	Immune response_IFN-alpha/beta signaling via JAK/STAT	2.26E-05	MxB, STAT1, PML, IFNAR2, ISG20, TAP1 (PSF1)
**23**	Role of iNKT and B cells in T cell recruitment in allergic contact dermatitis	2.83E-05	ITK, STAT1, CD5, CD21, CD19

**24**	B-regulatory cells and tumor cells intercellular interaction	3.54E-05	STAT1, PD-1, CD28, CD5, PD-L1, CD80
**25**	Role of Bregs in attenuation of T and NK cells mediated anti-tumor immune responses	3.63E-05	PD-1, LAG3, CD28, PD-L1, CD80
